# The KW18 peptide acts as a dual antimicrobial and immunomodulatory therapeutic candidate in the context of antimicrobial resistance

**DOI:** 10.1007/s10096-026-05417-4

**Published:** 2026-02-03

**Authors:** Layza Sá Rocha, Ana Cristina Jacobowski, Eduarda Thiburcio, Ana Paula de Araújo Boleti, Carolina Oliveira Matos, Juliana Bueno Barra, Luciano Morais Lião, Danieli Fernanda Buccini, Octávio Luiz Franco, Marlon Henrique Cardoso, Maria Ligia Rodrigues Macedo

**Affiliations:** 1 Laboratório de Purificação de Proteínas e suas Funções Biológicas, Universidade Federal do Mato Grosso do Sul, Campo Grande, MS Brazil; 2 Laboratório de RMN, Universidade Federal de Goiás, Goiânia, GO Brasil; 3 Programa de Pós-Graduação em Biotecnologia, S-Inova Biotech, Universidade Católica Dom Bosco, Campo Grande – MS, Campo Grande, MS Brazil; 4 Centro de Análises Proteômicas e Bioquímicas, Programa de Pós-graduação em Ciências Genômicas e Biotecnologia, Universidade Católica de Brasília, Brasília, DF Brazil; 5 Programa de Pós-Graduação em Ciências Ambientais e Sustentabilidade Agropecuária, Universidade Católica Dom Bosco, Campo Grande, MS Brazil

**Keywords:** Antimicrobial peptide, Antifungal activity, Anti-inflammatory activity, Host–pathogen interaction

## Abstract

**Purpose:**

Healthcare-associated infections and antimicrobial resistance present major challenges worldwide, especially for immunocompromised patients. This study aimed to evaluate the antimicrobial, antibiofilm, and immunomodulatory properties of KW18, a synthetic peptide (NH₂-KWIRRIIRRYVCFFRKFI-COOH) designed through bioinformatics to target multidrug-resistant bacteria and fungi, and to determine whether KW18 could serve as a dual-action therapy with potent microbicidal and anti-inflammatory effects.

**Methods:**

KW18 was tested against Gram-positive and Gram-negative bacteria (Staphylococcus aureus, Pseudomonas aeruginosa) and fungi (Candida glabrata, C. tropicalis). MIC and MBC assays, time-kill curves, and checkerboard synergy tests were performed. Its antibiofilm activity was evaluated on mature biofilms. Membrane disruption was assessed using Sytox™ Green and ergosterol-binding assays. Immunomodulatory effects were analyzed in LPS-stimulated BV-2 microglial cells by measuring nitric oxide, IL-6, and IL-10 levels, and cytotoxicity was evaluated with cell viability assays.

**Results:**

KW18 exhibited potent antimicrobial activity, with low MIC and MBC values across all strains, and retained antifungal effects even in the presence of ergosterol or sorbitol. Time-kill studies showed rapid, concentration-dependent microbicidal effects. The peptide worked synergistically with ciprofloxacin and amphotericin B, effectively inhibiting and eliminating mature biofilms and, in some cases, outperforming conventional treatments. Mechanistic tests confirmed membrane disruption. In BV-2 cells, KW18 reduced nitric oxide and IL-6 while increasing IL-10, all while maintaining over 85% cell viability, with a lower IC₅₀ for NO suppression than dexamethasone.

**Conclusion:**

KW18 demonstrates strong antimicrobial, antibiofilm, and immunomodulatory activities, supporting its potential as a promising dual-function therapeutic candidate for resistant infections.

## Introduction

 Healthcare-associated infections (HAIs), known as nosocomial infections, pose a significant global health challenge by prolonging hospital stays, increasing healthcare costs, and raising mortality rates [1, 2]. This issue is compounded by antimicrobial resistance (AMR), which is one of the top ten international health threats according to the World Health Organization (WHO) [3]. The misuse of antibiotics during the COVID-19 pandemic has intensified AMR, with 75% of hospitalized patients receiving antibiotics despite only 8% having confirmed bacterial infections [4]. Resistance rates for pathogens like *Staphylococcus aureus* have reached 35% in 76 countries, and multidrug-resistant infections caused by *Pseudomonas aeruginosa* and *Candida spp. have increased by more than* 20% globally from 2020 to 2022 [5]. Fungal pathogens, including *Candida glabrata* and *Candida tropicalis*, are particularly problematic for immunocompromised patients due to rising resistance to antifungal agents such as azoles and echinocandins [6].

In the 2025 update, the WHO identified *S. aureus*, carbapenem-resistant P. aeruginosa, and Candida spp. as high-priority threats [3]. These pathogens cause difficult-to-treat infections, including bloodstream, melanoma, and skin infections, and possess characteristics such as biofilm formation and notable resistance to antifungal and antibacterial agents [7]. Consequently, developing alternative therapies is essential 7,8]. Antimicrobial peptides (AMPs) are promising because of their broad-spectrum effectiveness, quick membrane disruption, action on intracellular targets, and reduced propensity for resistance development [8]. Recent advances in computational biology enable the rational design of improved AMPs with higher selectivity and stability, as well as additional properties, including anti-inflammatory and anticancer benefits [9]. Nonetheless, designing peptides that not only exhibit potent antimicrobial activity against priority pathogens but also reduce inflammation-related tissue damage remains a significant obstacle in peptide-based therapeutic development [8, 9].

This study introduces KW18, a synthetic multifunctional AMP designed using bioinformatics techniques to combat melanoma, multidrug-resistant bacteria, and fungi [10]. In a previous study by our research group, computational approaches were employed for sequence alignment, motif identification, and structural optimization, enabling the rational design and evaluation of peptide candidates [10]. Predictive analyses of antimicrobial activity were also conducted using physicochemical parameters such as net charge, amphipathicity, hydrophobic moment, and protein-binding potential [8–10]. Therefore, herein we evaluate the antibacterial, antifungal, and anti-inflammatory activities of KW18 against multidrug-resistant clinical pathogens, determine its three-dimensional structure, and aim to provide mechanistic insights and support its potential as a multifunctional therapeutic candidate.

## Materials and methods

### In Silico design

The KW18 peptide was developed through an integrative in silico pipeline to create a multifunctional peptide with antimicrobial, anticancer, and immunomodulatory capabilities. Initially, peptide sequences comprising 10 to 20 amino acids were extracted from specialized databases, including APD, DRAMP, and DBAASP, with a focus on peptides reported to exhibit antimicrobial and anticancer activities [9]. Sequence optimization used the Joker algorithm to preserve essential functional domains while enhancing physicochemical properties, including net positive charge, amphipathicity, hydrophobic moment, and selectivity [8, 10]. This automated and systematic design approach facilitated the selection of KW18 as a peptide candidate that is stable, selective, and biologically active [10].

### Nuclear magnetic resonance (NMR) spectroscopy and structure determination

The NMR sample was prepared by dissolving 1 mM of KW18 in a 40% (v/v) solution of 2,2,2-Trifluoroethanol (TFE-d3) in H₂O. DSS-d6 3-(Trimethylsilyl)−1-propanesulfonic acid sodium salt was used for internal reference. All spectra were recorded at 25 °C on a Bruker Avance III 500 spectrometer equipped with a 5 mm broadband inverse (BBI) probehead. The 1 H–1 H TOCSY experiment was acquired using the dipsi2gpph19 [11, 12] pulse sequence with 112 transients of 2048 × 256 points (F2, F1) and a spinlock mixing time of 70 ms. The 1 H-1 H NOESY experiment was acquired using the noesygpph19 pulse sequence with 104 transients, 4096 × 512 points, and a mixing time of 150 ms. The 1 H-13 C HSQC experiment was acquired using the hsqcetgp pulse sequence with 100 transients, 4096 × 512 points, and a 1-second relaxation delay (d1). All NMR data were processed and analyzed with nmrPipe [12] and CcpNmr Analysis [13], both available on the NMRbox platform [14]. The three-dimensional structures of KW18 were calculated using the Aria software version 2.3 in conjunction with CNS 1.2 [15, 16]. The intensities and volumes, classified as strong, medium, and weak based on NOE peak correlations in the NOESY spectrum, were semi-quantitatively converted into distance restraints using 1.72 Å, 3.2 Å, and 8.0 Å as the lower limit, reference distance, and upper limit, respectively. The amino acid sequence, chemical shift lists, dihedral angle values, and NOESY datasets served as input files for the structural determination process. Ultimately, 200 structures were generated for each of the 8 iterations, and the 10 lowest-energy structures resulting from water refinement were reported as representative of the KW18 conformational ensemble in solution. The structural quality was assessed using root-mean-square deviation (RMSD) values. Stereochemical quality was evaluated using the RamPlot web server [16] via the Ramachandran diagram, and fold-quality Z-scores were obtained from ProSA (Protein Structure Analysis) [16, 17]. Visualization, analysis, and manipulation of the three-dimensional structures were performed using PyMOL [17].

### Organism culture

To evaluate the antimicrobial activity of the KW18 peptide, commercially acquired fungi and bacteria catalogued in the American Type Culture Collection (ATCC) were used, following Clinical and Laboratory Standards Institute (CLSI) M07-A9 protocols. Among the tested bacteria were Gram-positive *S. aureus (ATCC 29213 and MRSA 43300)*,* Staphylococcus epidermidis (ATCC 00197)*,* Staphylococcus saprophyticus (ATCC 49453)*,* Staphylococcus haemolyticus (ATCC 29970)*,* Bacillus cereus (ATCC 11778)*, and *Enterococcus faecalis (ATCC 29212).* The Gram-negative bacteria tested comprised *A. baumannii (ATCC 19906)*,* Escherichia coli (ATCC 35218 and EHEC 43895)*,* Enterobacter aerogenes (ATCC 13048)*,* Enterobacter cloacae (ATCC 13047)*,* Klebsiella pneumoniae (ATCC 700603)*,* Klebsiella oxytoca (ATCC 13182)*,* P. aeruginosa (ATCC 27853)*,* Proteus mirabilis (ATCC 51286)*,* Serratia marcescens (ATCC 13880)*, and *Salmonella enterica* (ATCC 51741).

Included in the tested fungi and yeast strains were *Candida albicans* (ATCC 90028 and MYA 2876), C. *glabrata (ATCC 1707 and 90030)*,* Candida guilliermondii (ATCC 6260)*,* Candida krusei (ATCC 6258)*,* Candida parapsilosis (ATCC 22019)*,* C. tropicalis (ATCC 750)*,* Candida utilis (ATCC 9950)*,* Candida nivariensis (ATCC 9983)*,* Candida bracarensis (ATCC 10154)*, and *Cryptococcus gattii* (AFLP4). The microorganisms were stored at −20 °C in Brain Heart Infusion (BHI) broth supplemented with 20% glycerol. The assays were reactivated in BHI broth and incubated at 37 °C for 24 h (48 h for *C. gattii*). Plate cultures were performed using Mueller-Hinton agar (MHA) for bacteria and Sabouraud Dextrose agar (SDA) for fungi. Broth assays were conducted using Mueller-Hinton Broth (MHB), RPMI 1640, and BHI, adjusted according to experimental conditions.

### Determination of minimum inhibitory concentration (MIC)

KW18 was dissolved exclusively in sterile ultrapure water and used in all experimental assays. The minimum inhibitory concentration (MIC) was determined using the broth microdilution method in 96-well microplates, with two-fold serial dilutions ranging from 64 to 0.125 µM, following CLSI guidelines M07-A11 for bacteria and M27-S4 for fungi [18, 19]. Bacterial and fungal inocula were standardized to optical densities of 0.08–0.1 (595 nm for bacteria and 620 nm for fungi), corresponding to approximately 0.5 McFarland. Plates were incubated at 37 °C for 24 h for bacterial cultures and 48 h for fungal cultures. Ciprofloxacin and amphotericin B were used as positive controls, while wells containing only culture medium and microorganisms served as negative controls. All experiments were performed in triplicate and independently repeated three times.

### Determination of minimum bactericidal and fungicidal concentrations (MBC/MFC)

The minimum bactericidal concentration (MBC) and minimum fungicidal concentration (MFC) of the KW18 peptide were determined according to the Clinical and Laboratory Standards Institute (CLSI) guidelines, following protocols M07-A11 for bacteria and M27-S4 for fungi [18, 19]. After determining the MIC values, 10 µL aliquots were carefully collected from each well showing no visible microbial growth in the microdilution assays and plated onto Mueller–Hinton Agar (MHA) for bacterial strains and Sabouraud Dextrose Agar (SDA) for fungal strains. The inoculated plates were then incubated at 37 °C under aerobic conditions for 24 h for bacterial species and Candida spp., and for 48 h for Cryptococcus gattii, to accommodate its slower growth rate. After incubation, plates were examined for the presence or absence of visible colonies. The MBC/MFC was defined as the lowest peptide concentration that completely inhibited colony growth, confirming the bactericidal or fungicidal effect. Ciprofloxacin (for bacteria) and amphotericin B (for fungi) were used as positive controls. At the same time, wells containing only the microbial suspension in culture medium, without peptide treatment, served as negative growth controls. All experiments were performed in triplicate, with three independent repetitions to ensure reproducibility and statistical reliability of the results.

### Time-kill assay

The bactericidal and fungicidal kinetic assays were conducted using a time-dependent killing method adapted from Mitic-Culafic (2005) to evaluate the antimicrobial activity of the peptide KW18 [20]. The assays were performed against Staphylococcus aureus ATCC 29,213, Pseudomonas aeruginosa ATCC 27,853, Candida glabrata ATCC 1707, and Candida tropicalis ATCC 750. Microbial inocula were prepared by direct growth in appropriate culture media, and the cell density of bacterial and fungal suspensions was standardized by adjusting the optical density (OD) to 0.08–0.10 arbitrary units (AU), measured at 595 nm for bacterial cultures and at 620 nm for fungal cultures. Standardized suspensions were exposed to the peptide at concentrations corresponding to the minimum bactericidal concentration (MBC) for bacteria and the minimum fungicidal concentration (MFC) for fungi. Ciprofloxacin and amphotericin B were used as reference antimicrobial agents for bacterial and fungal growth, respectively, while untreated suspensions served as growth controls. At predetermined time points (0, 15, 30, 45, 60, 90, 120, 150, 180, 210, and 240 min), aliquots were aseptically withdrawn from each treatment and serially diluted when necessary. The samples were plated onto Mueller–Hinton agar (MHA) for bacterial strains and onto Sabouraud dextrose agar (SDA) for fungal strains, then incubated at 37 °C under appropriate conditions. After incubation, the number of viable microorganisms was determined by counting colony-forming units (CFU). The reduction in CFU over time was used to assess the peptide KW18’s time-dependent bactericidal and fungicidal effects. All experiments were carried out in triplicate, with three independent biological replicates, ensuring the reproducibility and reliability of the results.

### Checkerboard assay

The combined effect of the KW18 peptide with ciprofloxacin or amphotericin B was evaluated using checkerboard broth microdilution in 96-well plates, following Brennan-Krohn [21]. Bacterial inocula were prepared from isolated colonies grown on MHA, whereas fungal inocula were derived from colonies on SDA, suspended in sterile 0.9% NaCl. Optical densities were adjusted to 0.08–0.1 at 595 nm for bacteria (1.5 × 10⁸ CFU mL^−1^) and 620 nm for fungi (1.5 × 10⁶ CFU mL^−1^), corresponding to 0.5 McFarland. The KW18 peptide and reference drugs were serially diluted to create concentration matrices and incubated at 37 °C for 24 h. Growth was visually assessed, and the fractional inhibitory concentration index (FICI) was calculated as FICI = (MIC of KW18 in combination/MIC of KW18 alone) + (MIC of drug in combination/MIC of drug alone). Interactions were classified as synergistic, additive, indifferent, or antagonistic [21].

### Biofilm analysis assays

#### Quantitative evaluation

##### Spectrophotometric method for biofilm inhibition using 0.5% crystal violet

Biofilm formation by *Staphylococcus aureus* ATCC 29,213, *Pseudomonas aeruginosa* ATCC 27,853, *Candida glabrata* ATCC 1707, and *Candida tropicalis* ATCC 750 was assessed using 0.5% crystal violet staining, following standard methodologies described by Filloux (2014), Cruz (2018) [22, 23]. Microbial suspensions were prepared to achieve specific optical densities, and 180 µL of brain heart infusion (BHI) broth supplemented with 1% glucose and 20 µL of inoculum were added per well. Treatments with KW18, ciprofloxacin (for bacteria), and amphotericin B (for fungi) were applied at concentrations equivalent to the MIC and 10×MIC. Plates were incubated at 37 °C for 24 h. After incubation, the biofilms were stained with 0.5% crystal violet, washed, solubilized with glacial acetic acid, and the optical density was measured at 550 nm using a Varioskan Lux microplate reader (Thermo Scientific). Each assay was performed in triplicate with three independent experimental repetitions.

##### Spectrophotometric method for biofilm eradication using 0.5% crystal violet

Biofilm eradication was evaluated following adapted protocols from O’Toole (1998), using the same microbial strains and inoculum preparation described above [24, 25]. After an initial 24 h incubation at 37 °C to allow biofilm formation, the culture medium was removed and replaced with 200 µL of BHI broth containing 1% glucose and the respective treatments at 1×MIC and 10×MIC concentrations, as described in Sect. 4.3.6.1. Following an additional 24 h incubation, wells were washed with sterile distilled water, stained with 125 µL of 0.5% crystal violet for 10 min, rewashed, and air-dried for 1 h. Biofilms were then solubilized with 150 µL of 30% glacial acetic acid, and the optical density was read at 550 nm using a Varioskan Lux reader (Thermo Scientific). All assays were performed in triplicate with three independent repetitions.

#### Qualitative evaluation

##### Qualitative biofilm viability by fluorescence microscopy

The inhibitory and eradication effects of KW18 on biofilms were qualitatively assessed by fluorescence microscopy using the LIVE/DEAD^®^ BacLight™ kit (Thermo Fisher Scientific, 2024) [25, 26]. Cell viability was determined by dual staining with SYTO 9 and propidium iodide (PI), which differentiates viable (green) from nonviable (red) cells. For biofilm inhibition assays, microbial suspensions were incubated in 96-well plates pretreated with fetal bovine serum (FBS) and sodium bicarbonate to promote initial cell adhesion, as described by O’Toole [24, 27]. KW18, ciprofloxacin (for bacteria), and amphotericin B (for fungi) were added simultaneously with the inoculum at concentrations corresponding to 1×MIC and 10×MIC. Plates were then incubated for 24 h at 37 °C to allow biofilm formation in the presence of the treatments. After incubation, non-adherent cells were removed, wells were washed with sterile distilled water, and the remaining attached cells were stained with the LIVE/DEAD BacLight™ solution. For biofilm eradication assays, mature biofilms were first allowed to form by incubating the microbial suspensions for 24 h at 37 °C [28]. After this period, the culture medium was carefully removed and replaced with fresh medium containing KW18 or the reference antimicrobials at 1×MIC and 10×MIC concentrations. Following another 24 h incubation, wells were washed to remove planktonic cells and stained with the LIVE/DEAD BacLight™ solution. Fluorescence images were captured using a Leica DM 2000 LED microscope, allowing visualization of the proportion of live and dead cells within the biofilm structures. All assays were performed in triplicate with three independent repetitions.

### Investigation of antifungal mechanisms of action

#### Osmotic protection assay with sorbitol

Broth microdilution assays were conducted in RPMI-1640 medium supplemented with 0.8 M sorbitol to investigate how sorbitol influences the antimicrobial activity of the KW18 peptide [29]. The MIC measurements adhered to the standard protocol for MIC determination [22, 23]. This study aimed to explore the peptide’s mechanism of action and whether it depends on the integrity of the fungal cell wall. The activity of the KW18 peptide was tested with and without sorbitol, an osmotic protectant. The experiments were performed in sterile 96-well microplates, incubated at 37 °C for 24 h, and repeated three times with independent biological replicates.

#### Ergosterol binding assay

To assess KW18’s interaction with fungal membrane sterols, MICs were measured both with and without ergosterol, following the protocol by Freires [14]. This test aimed to determine whether KW18’s antifungal activity is mediated by binding to fungal sterols, such as ergosterol, thereby shedding light on its mode of action. Ergosterol was dissolved in 10% DMSO and 1% Tween 80, then added to RPMI-1640 medium to reach a final concentration of 400 µg mL-1. The broth microdilution technique using 96-well plates was used. Fungal suspensions were treated with the KW18 peptide in media containing or lacking ergosterol and incubated at 37 °C for 24 h. All experiments were conducted in triplicate and repeated three times.

#### Evaluation of membrane integrity using Sytox™ green assays

##### Quantitative analysis of membrane permeabilization

Microbial membrane permeability was assessed using the Sytox™ Green fluorescence assay, following manufacturer instructions and the protocol by Mohanram [30]. Microbial suspensions in potassium phosphate buffer were incubated with Sytox™ Green, a dye that enters cells with damaged membranes. The KW18 peptide was applied at 30 × MIC, a deliberately high concentration used to ensure rapid and measurable membrane disruption, enabling more precise detection of permeability kinetics. Fluorescence was continuously monitored for 240 min using a Varioskan Lux microplate reader (Thermo Fisher Scientific) to assess changes in membrane integrity. Buffer-only suspensions served as negative controls. All tests were performed in triplicate, with three biological replicates, to ensure accuracy and reproducibility. This method enabled detailed kinetic analysis of membrane permeabilization caused by the KW18 peptide under controlled conditions.

##### Qualitative visualization of membrane disruption

Qualitative assessment of membrane permeability was carried out on microbial cultures grown in suitable media using the KW18 peptide at its MIC—the lowest concentration preventing visible growth—in sterile 96-well microplates (Corning, USA). After 24 h of incubation at 37 °C, 30 µM of Sytox™ Green dye (Thermo Fisher Scientific, S7020, USA) was added [31]. This dye specifically penetrates cells with damaged membranes, enabling visualization of membrane injury. Fluorescence imaging was performed using a Leica DM 2000 fluorescence microscope (Leica Microsystems, Germany) to assess membrane disruption qualitatively. All tests were performed in triplicate across three independent experiments, enabling a precise visual evaluation of KW18’s impact on microbial membrane integrity under controlled conditions.

### Microbiological assessment of neuroinflammation activity

#### Nitrite quantification and cell viability (MTT Assay) in BV-2 microglial cells treated with KW18

The possible anti-inflammatory effects of the KW18 peptide were examined in BV-2 murine microglial cells by measuring nitrite levels and cell viability. BV-2 cells were grown in DMEM (Gibco, USA) supplemented with 10% fetal bovine serum (FBS) and 1% penicillin-streptomycin, maintained at 37 °C in a 5% CO₂ atmosphere. When the cells reached 80–90% confluence, they were treated with KW18 at concentrations of 2–20 µM for 24 h. Nitrite, indicative of nitric oxide (NO) production, was quantified using the Griess reaction at 540 nm with a Varioskan Lux microplate reader (Thermo Fisher Scientific, USA) [32]. Cell viability was evaluated via the MTT assay at 570 nm [31]. Controls included unstimulated cells and LPS-stimulated cells as a model for inflammation. All experiments were conducted in triplicate and analyzed using one-way ANOVA followed by Tukey’s test (*p* < 0.05).

#### Cytokine profiling via ELISA

KW18’s anti-inflammatory activity was evaluated in BV-2 murine microglial cells by quantifying IL-1β, IL-6, and IL-10 levels using sandwich ELISA kits from PeproTech and Thermo Fisher Scientific, following the manufacturer’s instructions [33–35]. BV-2 cells (1 × 10⁶ cells mL⁻¹) were treated with 4 µM KW18, a concentration selected based on preliminary viability and dose-response assays indicating that it was sufficient to elicit measurable cytokine modulation without causing cytotoxicity. Cells were incubated for 24 h at 37 °C in 5% CO₂, after which supernatants were collected and stored at − 20 °C. Cytokine levels were measured via absorbance at 405 nm, with correction at 650 nm, using a Varioskan Lux microplate reader (Thermo Fisher Scientific, USA). Standard curves confirmed assay accuracy: 16–2000 pg mL^− 1^ for IL-6, 32–4000 pg mL^− 1^ for IL-1β, and 32–2000 pg mL^− 1^ for IL-10.

## Results

### Synthetic peptide KW18

KW18 (NH₂-KWIRRIIRRYVCFFRKFI-COOH) is a purposefully designed peptide known for its antimelanoma effects, which also showed strong antimicrobial predictions in silico. To assess the antimicrobial likelihoods, we applied multiple prediction platforms based on machine-learning classifiers and biophysical property analysis, scoring 0.998 (Support Vector Machine) and 0.956 (potential of the peptide). The sequence was first evaluated using the CAMPR3 server, which integrates different algorithmic models for peptide classification. The results indicated a high likelihood of antimicrobial activity, with scores of 0.998 for the SVM model, 0.956 for Random Forest, and 0.990 for Discriminant Analysis. Complementarily, the DBAASP database — which classifies peptides based on experimentally validated structural and functional parameters — also categorized the sequence as antimicrobial. In addition, analysis through the *Sense the Moment* platform, which estimates conformational stability and interaction propensity in hydrophobic environments relevant to membrane insertion, yielded a score of 1.64, supporting the peptide’s stability and its potential for effective interaction with bacteria.

These results indicate broad-spectrum activity, particularly against S. aureus, P. aeruginosa, C. glabrata, and C. tropicalis, which are pathogens often associated with infections in melanoma patients. The dual function of KW18 as both an antimelanoma and antimicrobial agent highlights its potential to improve treatment outcomes and patient survival by targeting tumor growth and controlling opportunistic infections. Physicochemical analysis showed a molecular weight of 2501.13 Da, a net charge of + 7, hydrophobicity of 56%, a GRAVY score of 0.033, and a Boman index of 2.75 kcal mol^− 1^ (indicators of stability, membrane interaction, and protein-binding ability). The peptide was produced by Aminotech (Sorocaba, Brazil) with 95% purity, verified by high-performance liquid chromatography (HPLC) and mass spectrometry.

### Nuclear magnetic resonance (NMR) spectroscopy and structure determination

**Fig. 1:**
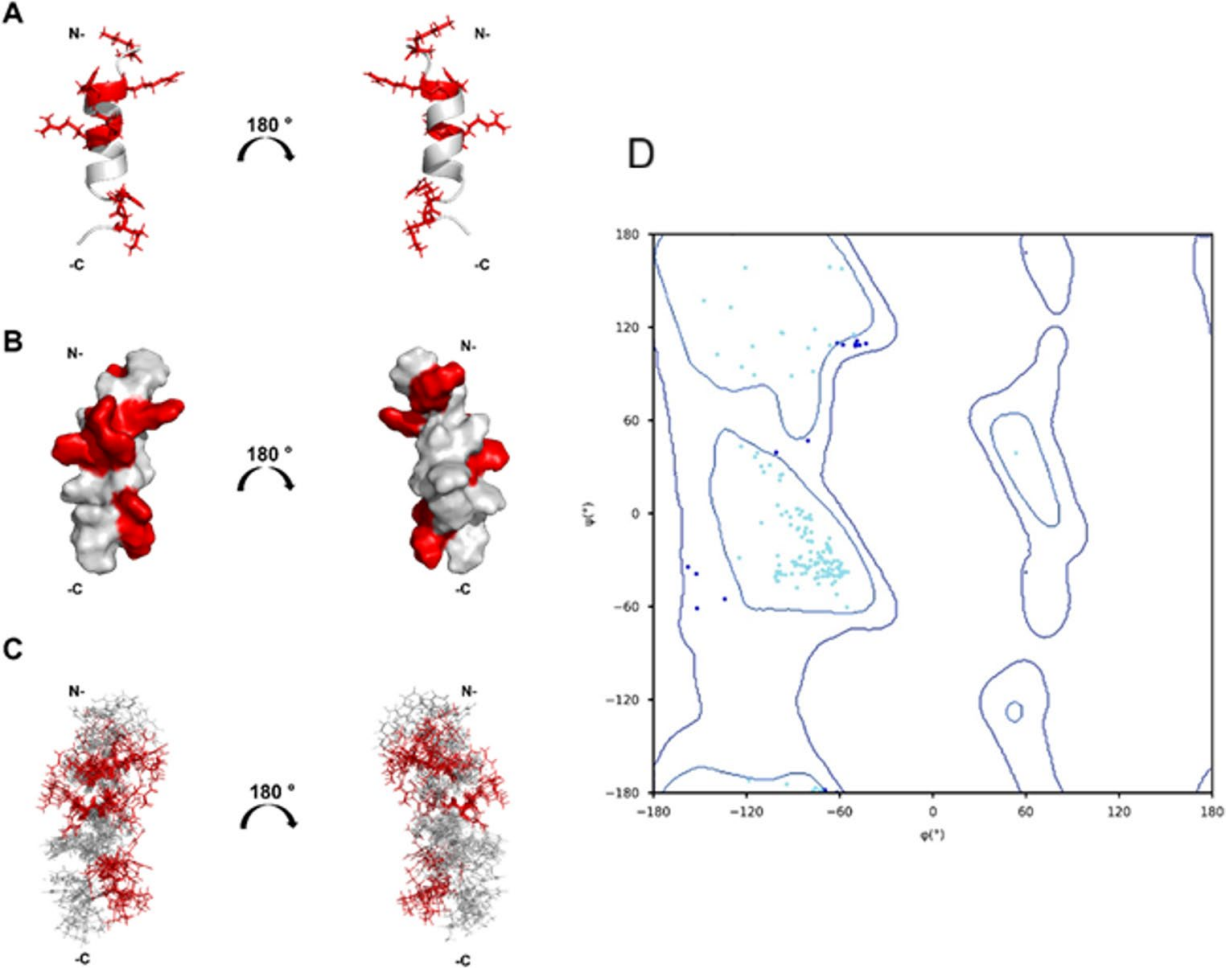
Solution NMR structures of KW18 in TFE-d_3_. (**A**) A ribbon representation of the lowest energy structure (hydrophobic residues in gray, cationic residues in red). (**B**) Electrostatic surfaces, red indicates positively charged residues. (**C**) Superposition of the 10 lowest-energy structures. All structures show a 180° inversion angle. (**D**) Validation of the 10 lowest-energy solution NMR structures of GWA17 using the Ramachandran plot. The graphic shows that 99.3% of residues were in the favored regions (cyan) of the plot, 0.7% were in the allowed regions (blue dots), and 0% were in the disallowed region (red)

NMR spectroscopy was employed to determine the three-dimensional structure of KW18 in 40% TFE-d₃/H₂O solution, to investigate structural characteristics potentially associated with its biological activity. Approximately 80% of the ^1H and ^13 C resonances were assigned using ^1H–^1H TOCSY, ^1H–^1H NOESY, and ^1H–^13 C HSQC spectra, enabling both backbone and side-chain NMR assignments of KW18 (Table 1). A total of 217 NOE distance restraints were identified—163 intra-residue and 34 inter-residue—alongside 28 dihedral angle restraints predicted by DANGLE. Structure calculations were carried out using ARIA 2.3 in combination with CNS 1.2. A total of 200 structures were generated, 200 conformers per iteration (it0-it8). The 20 lowest-energy structures from the final iteration (it8) were refined in water, and the 10 best representatives were selected to compose the final conformation ensemble of KW18. The three-dimensional structure of KW18 in 40% (v/v) TFE-d₃/H₂O revealed a well-defined α-helical conformation spanning residues Arg4 to Phe13 (Fig. 1A), in agreement with the CD data (data not shown).

**Table 1:** Presents the chemical shifts (in ppm) assigned to the hydrogens and carbons of each residue of the KW18 peptide, obtained by NMR spectroscopy. The columns correspond to the following assignments: H (amide hydrogens), HA (alpha hydrogens), HB (beta hydrogens), HG (gamma hydrogens), HD (delta hydrogens), HE (epsilon hydrogens), and HZ (zeta hydrogens, when applicable). The columns CA, CB, CG, CD, and CE correspond to the chemical shifts of the alpha, beta, gamma, delta, and epsilon carbons, respectively. Duplicate values indicate geminal or chemically equivalent hydrogens or carbons

Residue	H	HA	HB	HG	HD	HE	HZ	CA	CB	CG	CD	CE
Lys1		3.96	1.83, 1.83	1.38, 1.38	1.65, 1.65	2.93, 2.93		55.99	33.44	24.04	29.18	42.08
Trp2	8.56	4.79	3.27, 3.27		7.22			57.38	29.46		126.78	
Ile3	7.74	4.04	1.74	1.10, 0.82	0.87			61.79	38.84	27.54, 17.19	12.51	
Arg4	7.86	4.03	1.70, 1.70	1.52, 1.52	3.11, 3.11	7.14		57.85	30.46	27.26	43.60	
Arg5	7.76	4.05	1.67, 1.67	1.46, 1.46	3.08, 3.08	7.18		57.82	30.32	27.16	43.40	
Ile6	7.71	4.02	1.90	1.15, 0.86	0.82			62.66	38.47	27.71, 17.00	12.31	
Ile7	7.85	3.95	1.83	1.15, 0.87	0.77			62.97	38.27	28.00, 17.24	12.30	
Arg8	7.97	4.12	1.80, 1.80	1.56, 1.56	3.11, 3.11	7.14		57.84	30.52	27.12	43.34	
Arg9	7.82	4.15	1.79, 1.79	1.60, 1.60	3.09, 3.09	7.18		57.36	32.39	26.89	43.22	
Tyr10	7.87	4.47	3.21, 3.07					59.33	39.59			
Val11	7.92	3.86	2.04	0.88, 0.88				64.06	32.21	20.91		
Cys12	7.84	4.30	2.86, 2.86					59.97	27.44			
Phe13	7.83	4.57	2.84, 2.84					57.95	38.50			
Phe14	8.07	4.61	2.81, 2.81					54.65	38.85			
Arg15	7.78	4.28	1.75, 1.75	1.58, 1.58	3.16, 3.16	7.11		55.80	30.86	27.41	43.47	
Lys16	7.76	4.16	1.63, 1.63	1.19, 1.19	1.55, 1.55	2.88, 2.88	7.47	56.83	32.56	24.51	28.88	42.16
Phe17	8.22	4.36	3.10, 3.10					60.40	38.46			
Ile18	8.16	3.96	1.83	1.36, 0.82	0.78			58.77	38.40	27.35, 16.88	12.68	

The electrostatic surface potential of KW18 is depicted in Fig. 1B, with cationic residues shown in red and hydrophobic residues in gray regions that align with typical AMP features (Fig. 1). Structural statistics indicated a reliable model, including. The ensemble of the ten lowest-energy structures displayed a high degree of convergence (Fig. 1C), particularly within the ordered region (Ile3–Lys16), with root-mean-square deviations (RMSD) of 0.55 ± 0.17 Å and 1.63 ± 0.23 Å for backbone and heavy atoms, respectively. When all residues (Lys1–Ile18) were considered, the RMSD increased to 0.96 ± 0.19 Å for the backbone and 2.13 ± 0.10 Å for all heavy atoms. The Ramachandran analysis, with a plot of the 10 lowest-energy structures for KW18 (Fig. 1D), shows that 90.6% of the angles φ and ψ are located in the most favored regions, while 9.4% are in the allowed regions (Table 1). These results indicate the high quality of the calculated structures. Additionally, the fold quality assessed by ProSA-web yielded a Z-score of −0.25, indicating that the model is within the typical range for experimentally determined structures of similar size, and suggesting the absence of significant structural problems.

Figure 1 Solution NMR structures of KW18 in TFE-d_3_. (A) A ribbon representation of the lowest energy structure (hydrophobic residues in gray, cationic residues in red). (B) Electrostatic surfaces, red indicates positively charged residues. (C) Superposition of the 10 lowest-energy structures. All structures show a 180° inversion angle. (D) Validation of the 10 lowest-energy solution NMR structures of GWA17 using the Ramachandran plot. The graphic shows that 99.3% of residues were in the favored regions (cyan) of the plot, 0.7% were in the allowed regions (blue dots), and 0% were in the disallowed region (red).

### Antimicrobial activity of KW18

#### Determination of MIC, MBC, and MFC values

The antimicrobial activity of KW18 was assessed using broth microdilution assays per CLSI guidelines, measuring MIC, MBC, and MFC values (Table 2). KW18 demonstrated broad-spectrum effectiveness against Gram-positive and Gram-negative bacteria and clinically relevant fungi. Among Gram-positive bacteria, *E. faecalis* (MIC = 2 µM) and *S. epidermidis* (MIC = 4 µM) showed high susceptibility, comparable or even superior to ciprofloxacin. Conversely, *K. oxytoca* and *P. mirabilis* exhibited lower sensitivity (MIC = 64 µM). KW18 inhibited both methicillin-sensitive and -resistant *S. aureus* (MIC = 8 µM; MBC = 64 µM) and was active against *P. aeruginosa* (MIC = 8 µM) along with other Gram-negative strains such as *E. coli* and *K. pneumoniae* (MIC = 4 µM). Against fungi, KW18 exhibited potent activity against clinically important yeasts, with MICs ranging from 1 to 16 µM. Notably, *C. guilliermondii* (MIC = 1 µM) was more sensitive than *C. albicans*, *C. tropicalis*, and *C. glabrata*, which were inhibited at 4–8 µM, including resistant strains. These findings suggest that KW18 is a promising broad-spectrum antimicrobial, especially against opportunistic pathogens affecting immunocompromised patients.

**Table 2:** Broad-spectrum antimicrobial activity of KW18, including MIC, MBC, and MFC values against bacteria and *Candida spp*

Gram-Positive Bacteria	KW18		Ciprofloxacin			
	MIC (µM)	MBC (µM)	MIC (µM)	MBC (µM)		
*Bacillus cereus* ATCC 11,778	4	64	0.5	16		
*Enterococcus faecalis* ATCC 29,212	2	64	0.125	32		
*Staphylococcus aureus* ATCC 29,213*	8	64	0.5	1		
*Staphylococcus aureus MRSA* ATCC 43,300*	8	64	2	2		
Staphylococcus epidermidis ATCC 00197*	4	64	0.5	0.5		
*Staphylococcus haemolyticus ATCC 29,970*	4	16	0.5	16		
Staphylococcus saprophyticus ATCC 49,453*	4	4	1	1		
**Gram-Negative Bacteria**						
Acinetobacter baumannii ATCC 19,606*	8	64	1	1		
*Enterobacter aerogenes ATCC 13,048*	4	8	1	2		
*Enterobacter cloacae ATCC 13,047*	8	32	8	16		
*Escherichia coli ATCC 35,218*	4	8	0.125	0.125		
*Escherichia coli EHEC ATCC 43,895*	32	32	1	2		
*Klebsiella pneumoniae ATCC 700,603*	4	8	0.125	1		
*Klebsiella oxytoca ATCC 13,182*	64	64	0.125	16		
*Proteus mirabilis ATCC 51,286*	64	64	8	8		
Pseudomonas aeruginosa ATCC 27,853*	8	32	0.5	8		
*Salmonella Entérica ATCC 51,741*	8	64	0.25	1		
Serratia marcescens ATCC 13,880*	8	4	2	16		
**Fungi/Yeasts** **RPMI Medium**	**KW18**		**Amphotericin B**			
	**MIC** **(µM)**	**MFC (µM)**	**MIC** **(µM)**	**MFC** **(µM)**		
Candida glabrata ATCC 1707*	8	8	0.125	0.125		
Candida tropicalis ATCC 750*	4	4	0.125	0.25		
Candida parapsilosis ATCC 22,019*	2	4	2	2		
Candida albicans ATCC 90,028*	16	32	4	4		
*Candida albicans ATCC 5314*	8	16	2	4		
*Candida krusei ATCC 6258*	4	8	4	8		
*Candida guillermondii ATCC 6260*	1	2	0.125	0.125		
*Cryptococcus gattii AFLP4*	8	16	2	4		

Asterisks (*) denote strains capable of biofilm formation and were chosen for further biofilm inhibition and eradication tests

#### Time kill assay

**Fig. 2:**
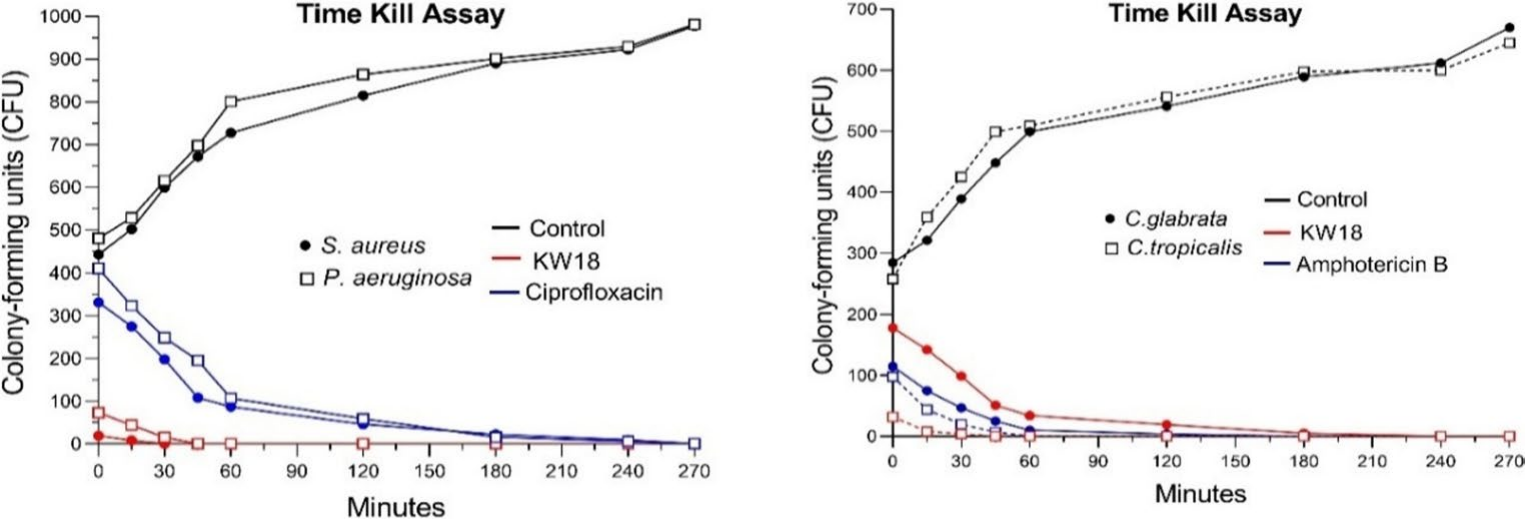
Time-kill kinetics of KW18 compared to standard antimicrobials against bacteria and fungi. The graph shows bacterial (left) and fungal (right) growth over 270 min after treatment with KW18 (red), standard drugs (ciprofloxacin or amphotericin B, blue), and a control (black). Solid circles (●) represent *S. aureus* and *C. glabrata*, while open squares (□) denote *P. aeruginosa* and *C. tropicalis*. Growth is measured in colony-forming units (CFU)

Some strains exhibited lower MIC and MBC values than conventional antimicrobial agents. However, the KW18 peptideKW18 demonstrated broad-spectrum activity against *S. aureus*,* P. aeruginosa*,* C. tropicalis*, and *C. glabrata* - pathogens highly relevant to melanoma-associated infections. This underscores its potential as a dual anticancer and antimicrobial agent, which is why it was selected for further studies. In time-kill assays, KW18 rapidly eradicated *S. aureus* within 30 min and *P. aeruginosa* within 45 min, outperforming ciprofloxacin. For fungi, *C. tropicalis* was eliminated in 45 min (faster than amphotericin B at 60 min), and *C. glabrata* in 240 min (slightly slower than amphotericin B at 180 min). These results emphasize KW18’s rapidKW18’s broad-spectrum activity against pathogens, which is especially important for immunocompromised melanoma patients (Fig. 2).

### Checkerboard assay

KW18 showed synergistic effects when used with standard antimicrobials. It demonstrated synergy against *S. aureus* ATCC 29,213 (FICI = 0.374) and *P. aeruginosa* ATCC 27,853 (FICI = 0.5), as well as with amphotericin B against *C. glabrata* ATCC 1707 (FICI = 0.28) and *C. tropicalis* ATCC 750 (FICI = 0.365) (Table 3). FICI values of 0.5 or below indicate synergy, values from > 0.5 to 1.0 show additivity, 1.0 to 4.0 indicate indifference, and above 4.0 suggest antagonism, based on CLSI guidelines. These findings imply that KW18 could enhance drug effectiveness by increasing membrane permeability or facilitating cellular uptake, particularly against Gram-negative bacteria and resistant fungi (Table 3).

**Table 3:** MICs of KW18 and standard drugs against bacterial and fungal strains

Strain	Combination	Individual MICs (µM)		Combined MICs (µM)		∑FICI	Activity
Bacteria		a	b	a	b	FICI_a_ + FICI_B_	
*S. aureus ATCC 29,213*	Pep (a) + ciprofloxacin (b)	8	0.5	2	0.062	0.374	Synergistic
*P. aeruginosa ATCC 27,853*	Pep (a) + ciprofloxacin (b)	8	0.5	2	0.125	0.5	Synergistic
Fungi	**Combination**	**c**	**d**	**c**	**d**	**FICI**_**c**_ **+ FICI**_**d**_	**Activity**
*C. glabrata ATCC 1707*	Pep (c) + Amphotericin B (d)	8	0.125	2	0,0037	0.28	Synergistic
*C. tropicalis ATCC 750*	Pep (c) + Amphotericin B (d)	4	0.125	0.5	0.03	0.365	Synergistic

For bacteria, “a” is KW18 and “b” is ciprofloxacin. For fungi, “c” is KW18 and “d” is amphotericin B. The combined MICs, the sum of fractional inhibitory concentrations (∑FICI), evaluate the agents’ interaction (Kmiecikowski, 2025)

### Biofilm analysis assays

#### Biofilm inhibition assay in bacteria and fungi

**Fig. 3:**
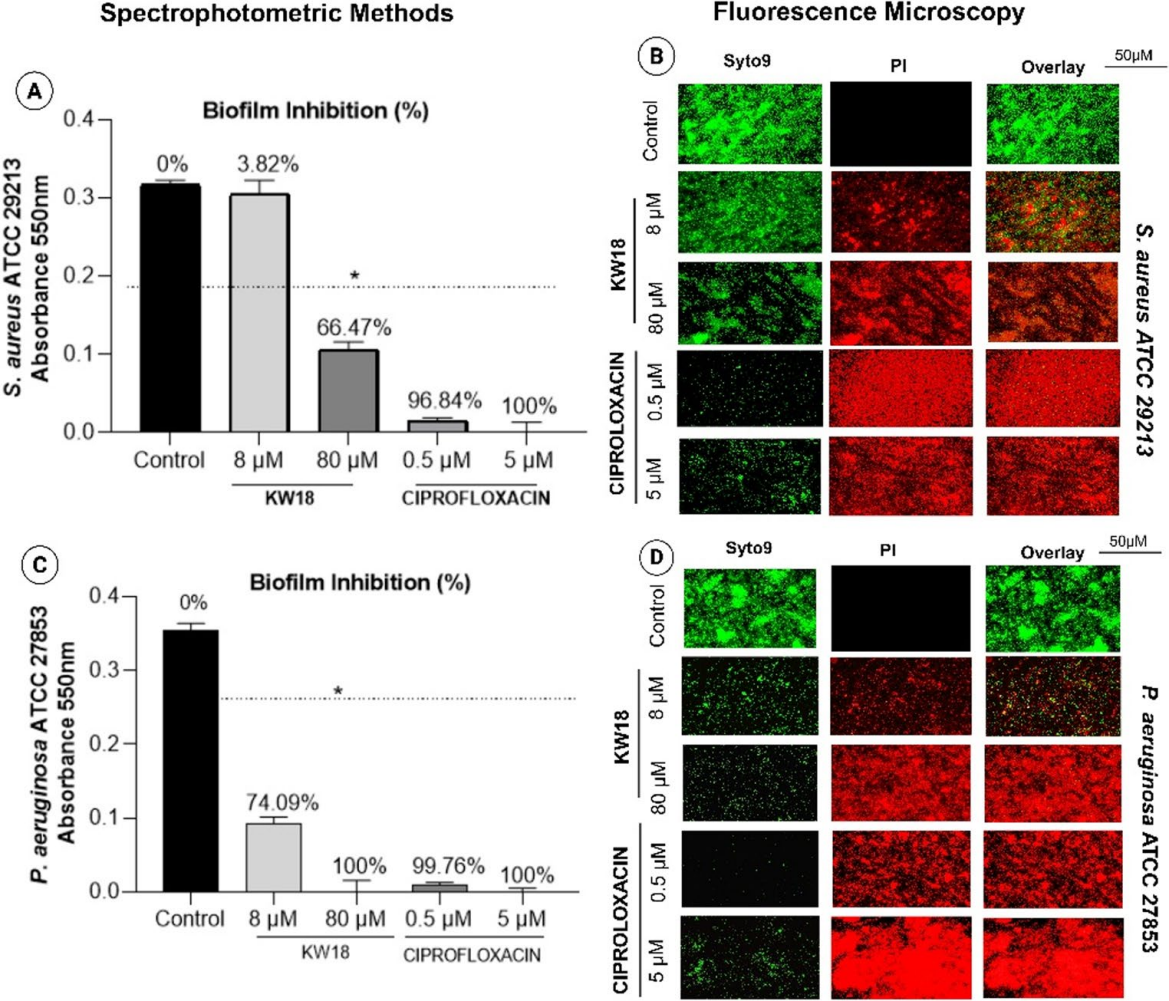
Biofilm Inhibition by the KW-18 peptide and ciprofloxacin: Quantitative and Qualitative Analysis. (**A**, **C**) Biofilm biomass by spectrophotometry (absorbance at 550 nm) after crystal violet staining for *S. aureus* (**A**) and *P. aeruginosa* (**C**). (**B**, **D**) Fluorescence microscopy with Syto9 (green, live cells) and PI (red, dead cells) shows reduced viability after KW18 and amphotericin B for *S. aureus* (**B**) and *P. aeruginosa* (**D**)—scale: 50 μm

The KW18 peptide demonstrated notable antibiofilm effects against *S. aureus* (ATCC 29213), *P. aeruginosa* (ATCC 27853), *C. glabrata* (ATCC 1707), and *C. tropicalis* (ATCC 750), as shown by crystal violet staining and fluorescence microscopy. At 80 µM, KW18 reduced *S. aureus* biofilm formation by 66.47%, whereas ciprofloxacin achieved 100% inhibition at 5 µM. For *P. aeruginosa*, KW18 caused a 74.09% reduction at 8 µM and inhibited biofilm formation at 80 µM, comparable to ciprofloxacin. Fluorescence microscopy supported these findings, mainly revealing non-viable cells within treated biofilms. These results indicate that KW18 is fully effective against *P. aeruginosa* biofilms. Quantitative and qualitative analyses of biofilm inhibition by the KW18 peptide and ciprofloxacin are presented in Fig. 3.

**Fig. 4:**
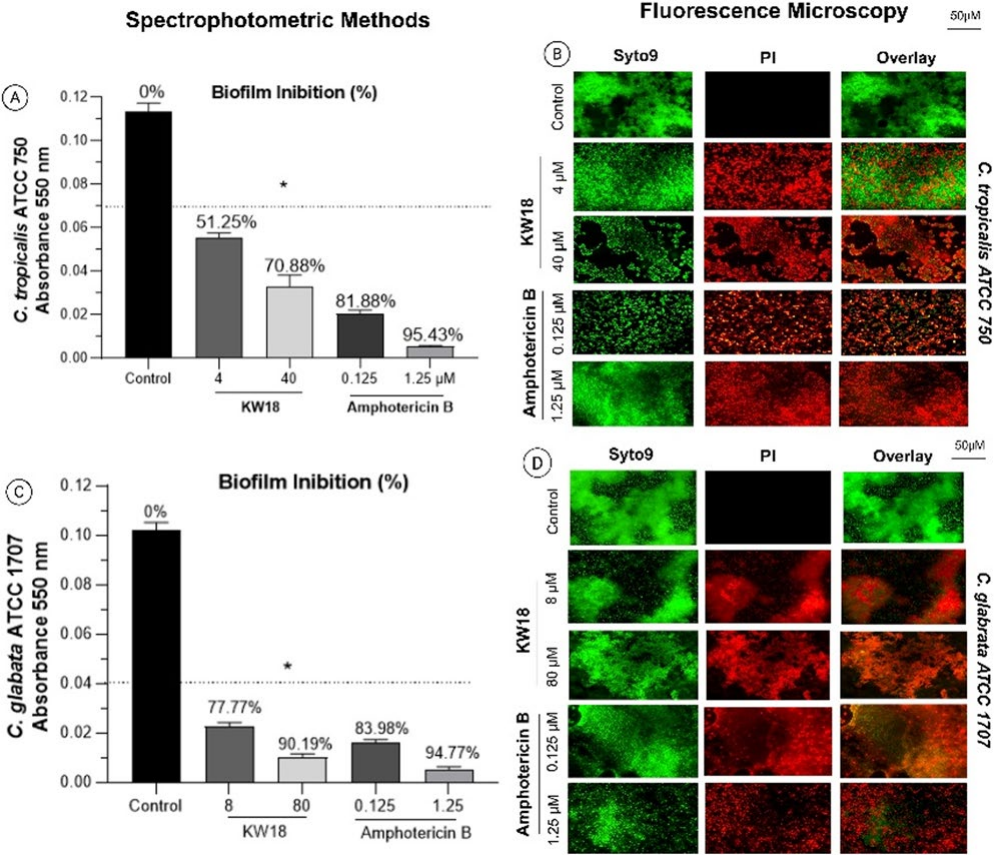
Biofilm Inhibition by KW18 and Amphotericin B—Quantitative and Qualitative Assessment. (**A**, **C**) Biofilm biomass measured by spectrophotometry (absorbance at 550 nm after crystal violet staining) for *C. tropicalis* (**A**) and *C. glabrata* (**C**). (**B**, **D**) Fluorescence microscopy using Syto9 (green, indicating live cells) and PI (red, indicating dead cells) demonstrates decreased viability following treatment with KW18 and amphotericin B for *C. tropicalis* (**B**) and *C. glabrata* (**D**)—scale: 50 μm

KW18 exhibited strong antibiofilm activity against *C. glabrata* and *C. tropicalis*, with 90.20% and 70.84% inhibition at high doses. While amphotericin B was more effective early, KW18 was comparable at higher doses, especially against *C. glabrata*. Fluorescence microscopy showed reduced cell viability, with a predominance of non-viable cells. These findings suggest that KW18 may be a potential treatment for fungal biofilms. Figure 4 presents biofilm inhibition analysis of KW18 and amphotericin B.

Figure 4 Biofilm Inhibition by KW18 and Amphotericin B—Quantitative and Qualitative Assessment. (A, C) Biofilm biomass measured by spectrophotometry (absorbance at 550 nm after crystal violet staining) for *C. tropicalis* (A) and *C. glabrata* (C). (B, D) Fluorescence microscopy using Syto9 (green, indicating live cells) and PI (red, indicating dead cells) demonstrates decreased viability following treatment with KW18 and amphotericin B for *C. tropicalis* (B) and *C. glabrata* (D)—scale: 50 μm.

#### Biofilm eradication assay in Pre-formed bacterial and fungal biofilms

**Fig. 5:**
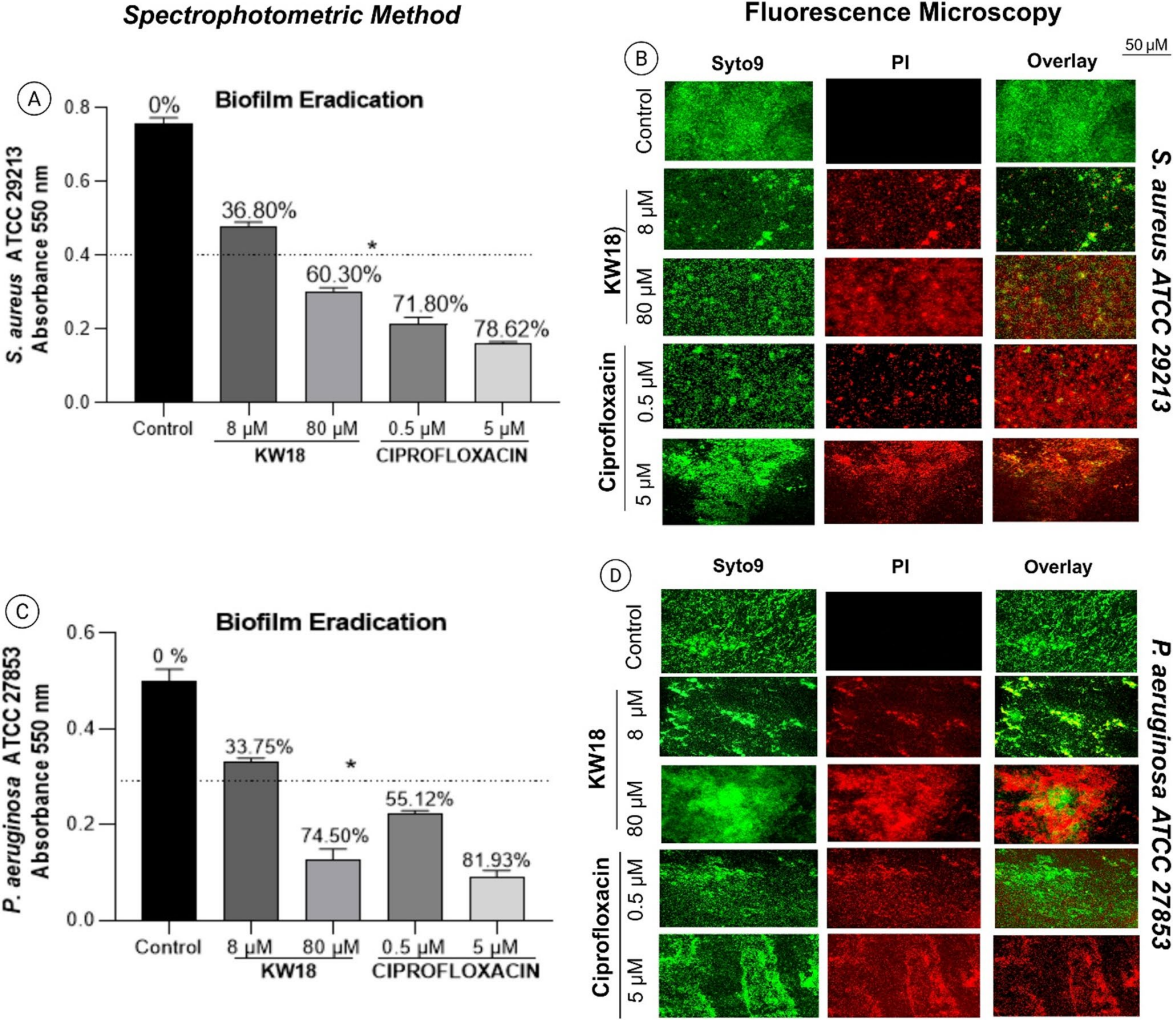
Bacterial biofilm eradication by the KW-18 peptide and ciprofloxacin. (**A**, **C**) Biofilm biomass was quantified with crystal violet staining and spectrophotometry at 550 nm for *S. aureus* (**A**) and *P. aeruginosa* (**C**). (**B**, **D**) Fluorescence microscopy visualized biofilms with Syto9 (green, living cells) and PI (red, dead cells) in treated and untreated *S. aureus* (**B**) and *P. aeruginosa* (**D**). Scale bar: 50 μm

The KW18 peptide effectively inhibits bacterial and fungal biofilms. It reduced *S. aureus* ATCC 29,213 biofilms by 60.30% at 80 µM, nearing ciprofloxacin’s 78.62% at 5 µM. For *P. aeruginosa* ATCC 27,853, KW18 achieved a 74.50% reduction at 80 µM, close to ciprofloxacin’s 81.93%. Fluorescence microscopy supported these findings by revealing more red-stained (non-viable) cells following treatment (Fig. 5).

Figure 5 Bacterial biofilm eradication by the KW-18 peptide and ciprofloxacin. (A, C) Biofilm biomass was quantified with crystal violet staining and spectrophotometry at 550 nm for *S. aureus* (A) and *P. aeruginosa* (C). (B, D) Fluorescence microscopy visualized biofilms with Syto9 (green, living cells) and PI (red, dead cells) in treated and untreated *S. aureus* (B) and *P. aeruginosa* (D)—scale bar: 50 μm.

**Fig. 6:**
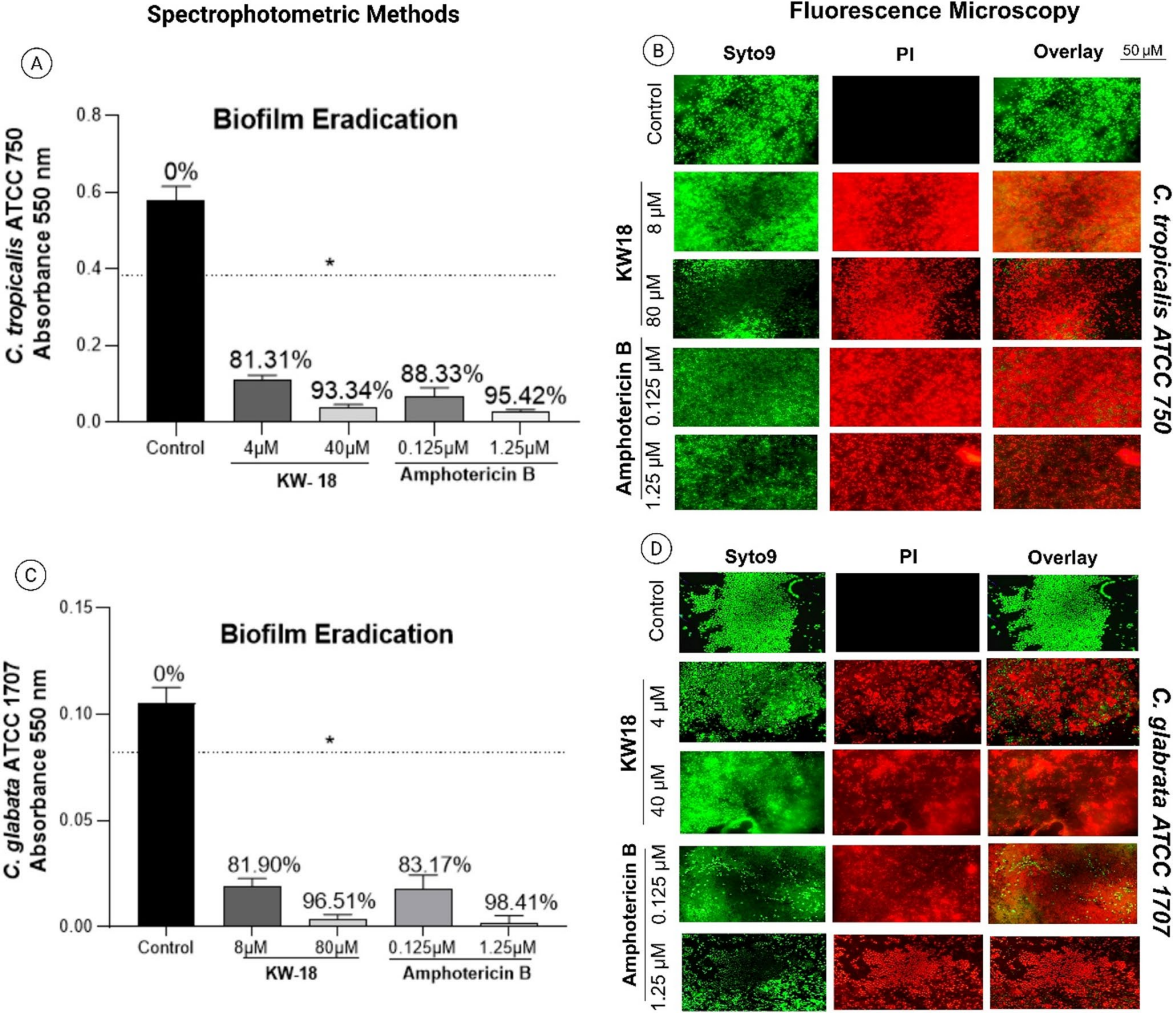
Fungal biofilm eradication by the peptide KW18 and amphotericin B. Quantitative biofilm analysis used crystal violet staining, measuring absorbance at 550 nm for *C. tropicalis* (**A**) and *C. glabrata* (**C**). Cell viability after treatment was assessed by fluorescence microscopy with Syto9 and PI for *C. tropicalis* (**B**) and *C. glabrata* (**D**). Scale bar: 50 μm

In fungal biofilm tests, KW18 significantly reduced biofilms, with 93.34% eradication of *C. tropicalis* ATCC 750 at 40 µM and 96.51% of *C. glabrata* ATCC 1707 at 80 µM. Amphotericin B was marginally more effective, achieving 95.42% and 98.41%, respectively. Fluorescence microscopy supported these findings, mainly revealing PI-stained cells in treated samples (Fig. 6). KW18 demonstrated strong potential for biofilm eradication across various bacterial and fungal strains. Although ciprofloxacin and amphotericin B showed slightly better results at lower doses, KW18’s efficacy at higher concentrations suggests its promise as an alternative or complement in biofilm infection treatment.

### Investigation of antifungal mechanisms of action

#### Osmotic protection assay with sorbitol

Adding sorbitol to the growth medium increased KW18’s antifungal activity against *C. tropicalis* ATCC 750, lowering the MIC to 1 µM and MFC to 2 µM, surpassing amphotericin B’s MIC of 2 µM and MFC of 4 µM. For *C. glabrata* ATCC 1707, KW18’s MIC was 8 µM and MFC 16 µM, aligning with ergosterol-binding assay results, while amphotericin B was more effective (MIC: 2 µM; MFC: 8 µM). Visual and spectrophotometric tests confirmed increased KW18 activity under osmotic stress, especially against *C. tropicalis*. KW18 and amphotericin B’s antifungal activity in sorbitol medium is shown in Table 4.

**Table 4:** KW18 and amphotericin B in sorbitol media

Fungi (Sorbitol Medium)	KW18		Amphotericin B	
	MIC (µM)	MFC (µM)	MIC (µM)	MFC (µM)
*Candida glabrata ATCC 1707*	8	16	2	8
*Candida tropicalis ATCC 750*	1	2	2	4

Under osmotic stress, KW18 is highly effective against *C. tropicalis* and *C. glabrata*, as shown by MIC and MFC

#### Ergosterol binding assay

In the presence of exogenous ergosterol, the KW18 peptide maintained moderate antifungal activity against Candida glabrata ATCC 1707, exhibiting an MIC of 8 µM and an MFC of 16 µM. Amphotericin B, as expected for an ergosterol-binding antifungal, showed greater potency under these conditions, with an MIC of 2 µM and an MFC of 4 µM. Against Candida tropicalis ATCC 750, KW18 displayed MIC and MFC values of 4 µM, while amphotericin B presented the same MIC but a higher MFC of 8 µM. The relative stability of KW18’s antifungal activity in the presence of ergosterol—particularly compared to the marked reduction observed for amphotericin B—suggests that KW18 does not rely on direct ergosterol binding for its mechanism of action. Instead, its fungicidal effect appears to result from general membrane perturbation rather than specific sterol interaction. These results, summarized in Table 5, indicate that KW18 retains antifungal efficacy in ergosterol-enriched environments, supporting the hypothesis of an ergosterol-independent mode of membrane disruption.

**Table 5:** KW18 and amphotericin B in ergosterol media

Fungi (Ergosterol Medium)	KW18		Amphotericin B	
	MIC (µM)	MFC (µM)	MIC (µM)	MFC (µM)
*Candida glabrata ATCC 1707*	8	16	2	4
*Candida tropicalis ATCC 750*	4	4	4	8

Ergosterol increases the efficacy of amphotericin B, as indicated by MIC and MFC results

### Evaluation of membrane integrity using Sytox™ green assays

**Fig. 7:**
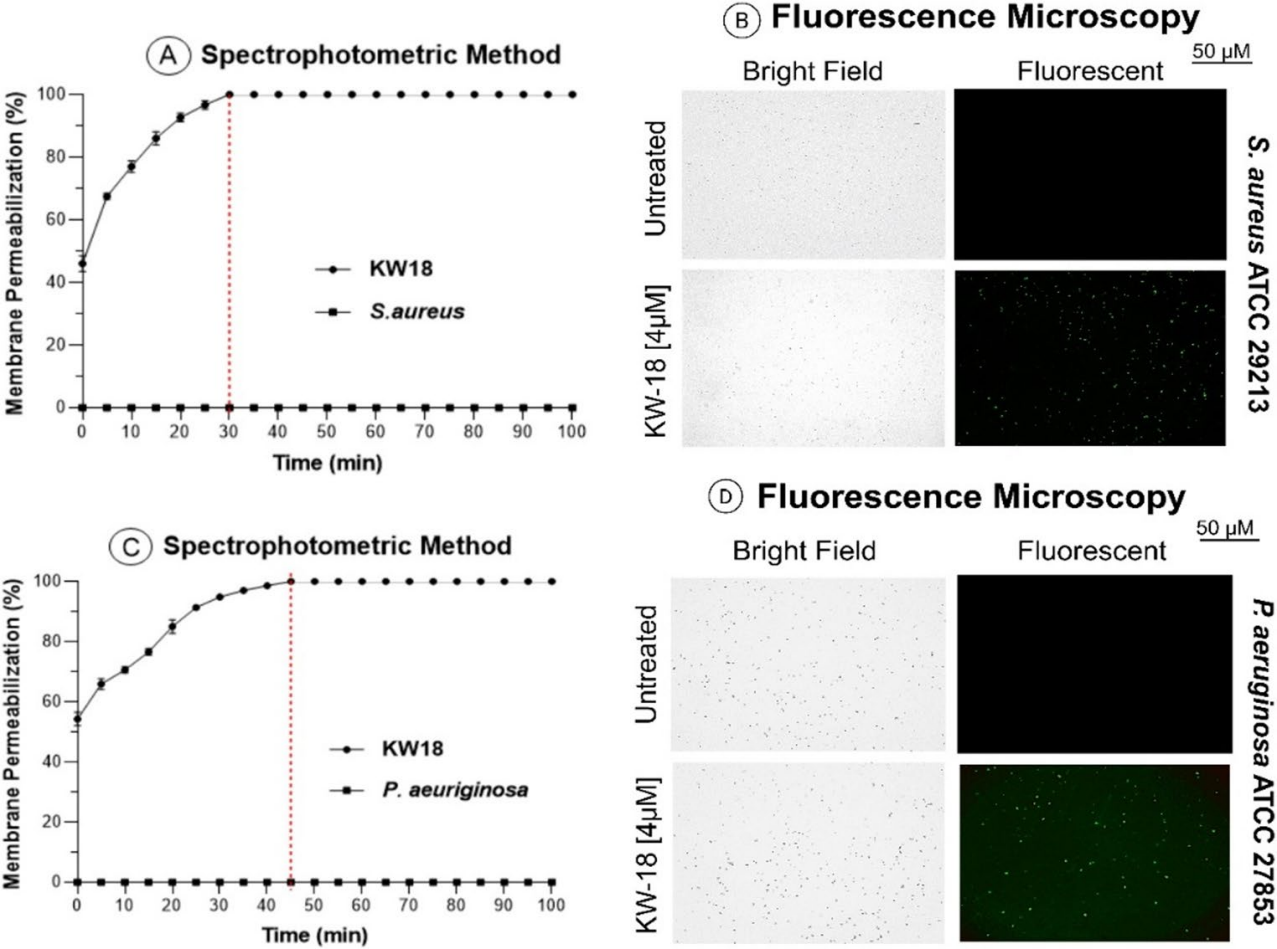
Bacteria membrane permeability testing with KW18. (**A**, **C**) Membrane permeabilization over time in *S. aureus* and *P. aeruginosa* treated with 4 µM KW18. (**B**, **D**) Fluorescence images verify membrane damage after peptide exposure

To demonstrate how KW18 functions, membrane integrity assays utilized Sytox™ Green, a fluorescent dye that penetrates cells with compromised membranes. Spectrophotometric kinetic assays revealed that KW18 rapidly permeabilizes *S. aureus* ATCC 29,213 membranes within 30 min and *P. aeruginosa* ATCC 27,853 within 45 min (Fig. 7A and C). This suggests a rapid action on Gram-positive and Gram-negative bacteria. Fluorescence microscopy confirmed that untreated cells exhibited no fluorescence, while treated cells displayed intense green fluorescence, indicating membrane disruption (Fig. 7B and D).

**Fig. 8:**
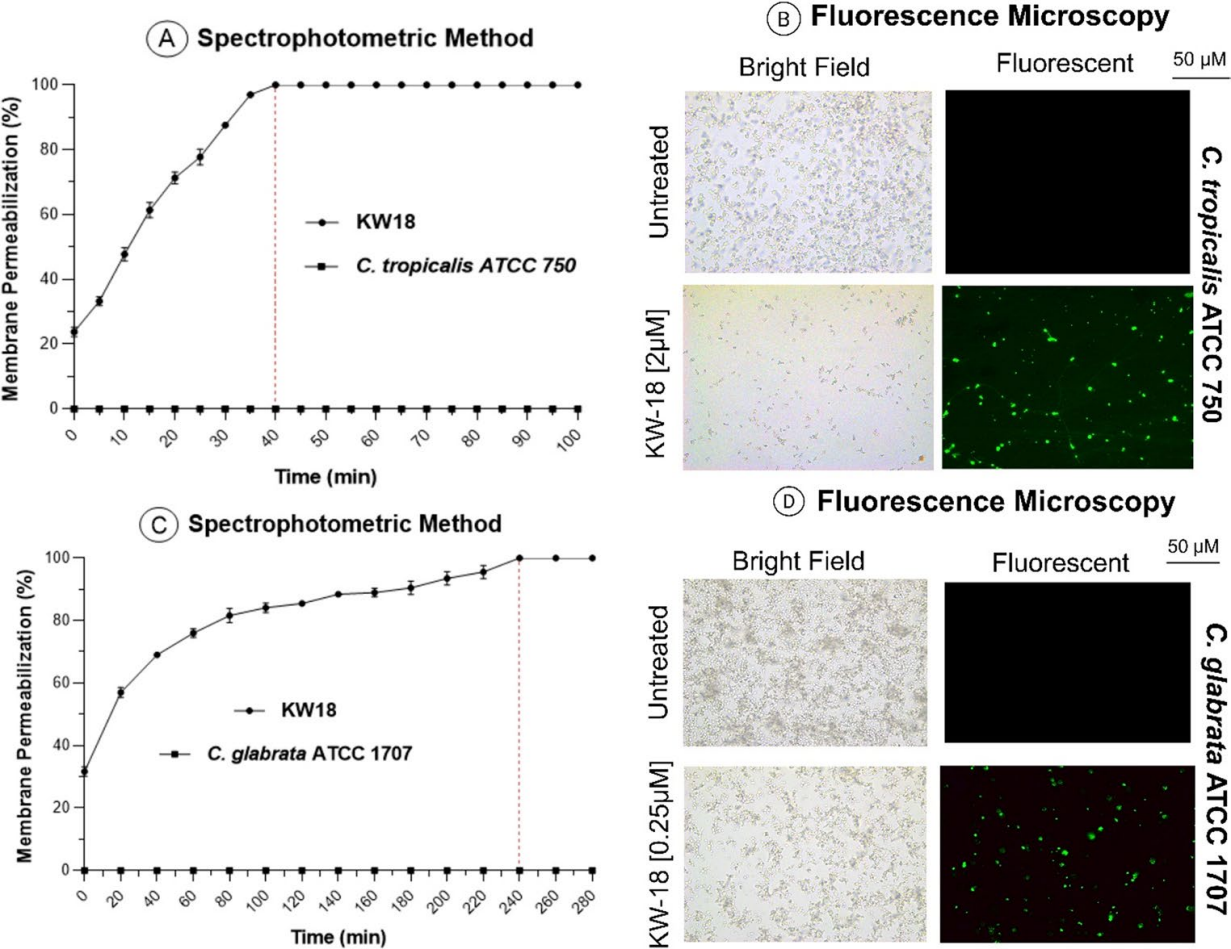
Plasma membrane permeabilization in *C. tropicalis* and *C. glabrata* following KW18 treatment in RPMI medium. Spectrophotometry assesses membrane integrity (**A**, **C**). Fluorescence microscopy reveals more permeabilization in *C. tropicalis* at lower concentrations and shorter exposure times than in *C. glabrata* (**B**, **D**)

Fungi Sytox™ Green assays on *C. tropicalis* ATCC 750 and *C. glabrata* ATCC 1707 revealed species-specific responses (Fig. 8). *C. tropicalis* was fully permeabilized (100%) within 45 min at 2 µM, whereas *C. glabrata* took 240 min and 4 µM to reach similar effects. These findings indicate a resistance profile in *C. glabrata*. Fluorescence microscopy confirmed this, showing strong fluorescence signals in treated cells, especially in *C. tropicalis*. The permeabilization of the plasma membrane in both species after KW18 treatment in RPMI medium is depicted in Fig. 8.

**Fig. 9:**
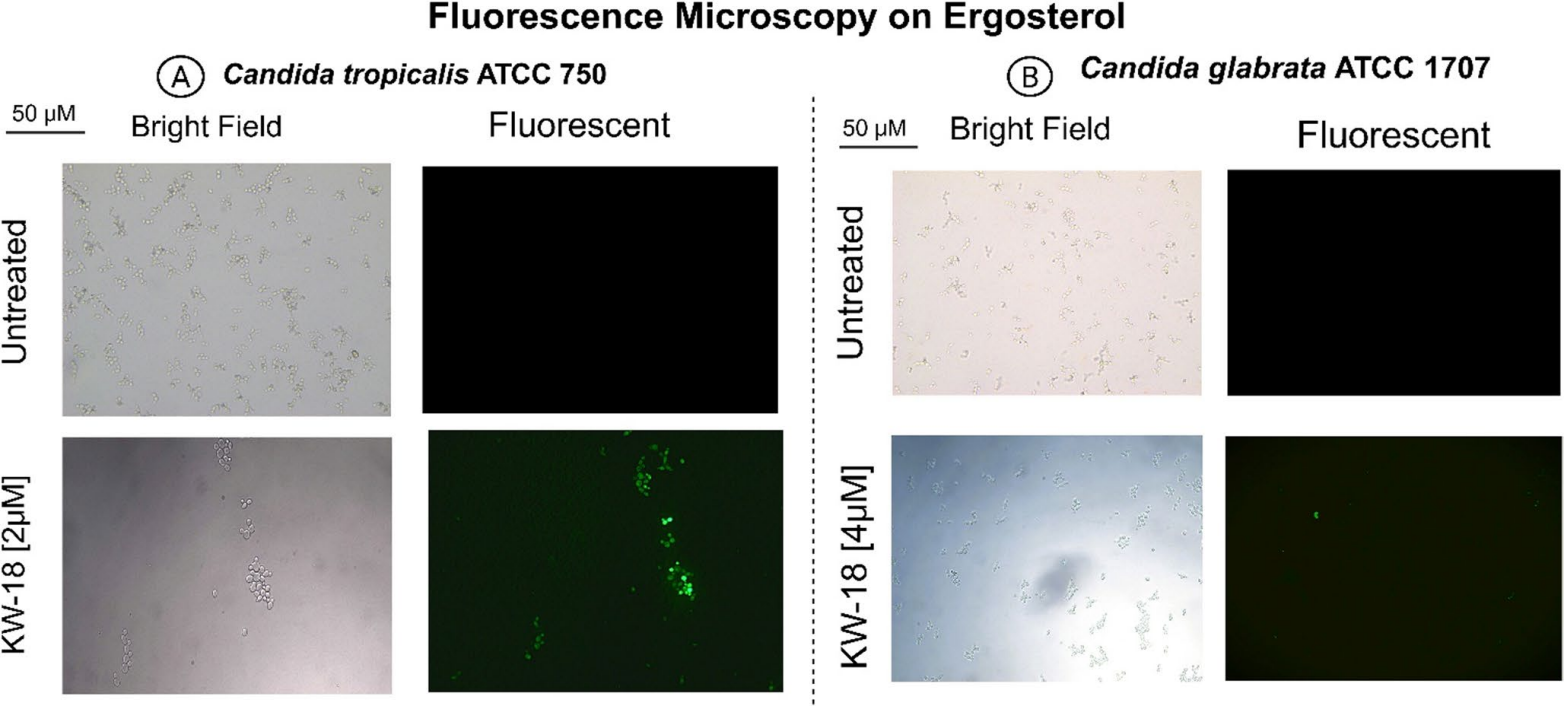
Fluorescence microscopy showing KW18 interacting with ergosterol in *C. tropicalis* and *C. glabrata*. (**A**) *C. tropicalis* and (**B**) *C. glabrata* treated with KW18 emit fluorescence, indicating peptide binding to ergosterol-rich membranes. No fluorescence was observed in untreated controls

To further investigate the potential interaction between KW18 and fungal membrane components, fluorescence microscopy assays were performed in RPMI medium supplemented with ergosterol. In control samples, no fluorescence was detected. Upon treatment with KW18 at 2 µM for *C. tropicalis* and 4 µM for *C. glabrata*, fluorescence signals indicative of membrane permeabilization were observed (Fig. 9). However, given that the presence of exogenous ergosterol did not substantially reduce the antifungal activity of KW18—particularly against *C. tropicalis*—these results suggest that the peptide’s mechanism of action is unlikely to depend on direct ergosterol binding. Instead, KW18 appears to exert its antifungal effect primarily through general membrane disruption driven by electrostatic and amphipathic interactions with the lipid bilayer. The observed increase in the MIC of amphotericin B in the presence of ergosterol, a well-known ergosterol-binding compound, served as an internal control validating the experimental setup. Together, the spectrophotometric and microscopy data indicate that KW18 destabilizes microbial membranes through a non-specific, ergosterol-independent mechanism, with differences in susceptibility likely reflecting species-specific variations in membrane composition and dynamics.

### Microbiological assessment of neuroinflammation activity

#### Nitrite quantification and cell viability (MTT Assay) in BV-2 microglial cells treated with KW18

**Fig. 10:**
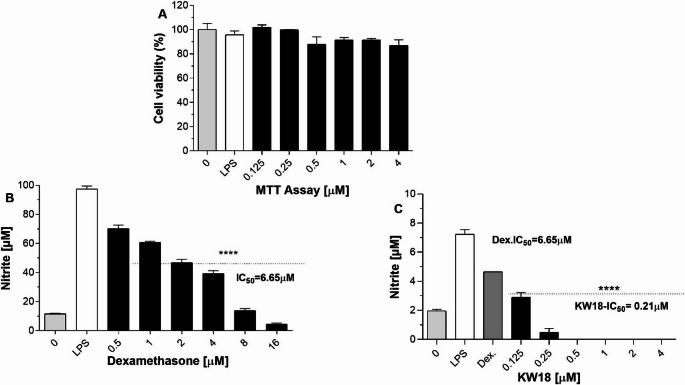
Cell viability and nitrite production in LPS-stimulated BV-2 microglial cells treated with KW18 or dexamethasone. (**A**) KW18 (0.125–4 µM) did not affect cell viability after 24 h, with MTT values consistently above 85%, demonstrating absence of cytotoxicity within the biologically relevant range. Concentrations above 4 µM were not included because preliminary tests indicated reduced solubility and potential interference with the MTT reaction, and the doses displayed in the figure were sufficient to elicit a clear biological response. (**B**) Dexamethasone (0.5–16 µM) reduced nitrite levels in a dose-dependent manner, reaching an IC₅₀ of 6.65 µM. A broader concentration range was applied because higher doses are required for this drug to exhibit measurable anti-inflammatory effects. (**C**) KW18 inhibited nitrite accumulation in a dose-dependent fashion, with a markedly lower IC₅₀ of 0.21 µM. As the curve demonstrated strong inhibition already within the lowest concentrations plotted, extending the graph beyond 4 µM was unnecessary, since all relevant activity was captured within this effective range and higher concentrations did not contribute additional information

To evaluate the anti-inflammatory effects of the synthetic peptide KW18 in a context relevant to the pathogens targeted in this study—including *S. aureus*, *P. aeruginosa*, *C. glabrata*, and *C. tropicalis*—BV-2 microglial cells were stimulated with 1 µg mL⁻¹ LPS and treated with increasing concentrations of KW18 (0.125–4 µM). Cell viability assessed by the MTT assay (Fig. 10A) remained above 85% across all concentrations, demonstrating that KW18 was not cytotoxic within this range. The upper limit of 4 µM was selected based on both technical and biological criteria. Concentrations above this threshold showed reduced solubility and signs of interference with the MTT reagent, compromising assay accuracy. Importantly, this interval includes the concentrations at which KW18 displayed antimicrobial activity against the primary target pathogens of this study: *S. aureus* (MIC = 8 µM), *P. aeruginosa* (MIC = 8 µM), *C. glabrata* (MIC = 8 µM), and *C. tropicalis* (MIC = 4 µM). Given that the graphical range tested already demonstrated measurable biological activity at low micromolar levels, and that the data clearly indicated no cytotoxicity within the relevant therapeutic window, extending the MTT assay to higher concentrations was not justified nor required.

Figure 10. Cell viability and nitrite production in LPS-stimulated BV-2 microglial cells treated with KW18 or dexamethasone. (A) KW18 (0.125–4 µM) did not affect cell viability after 24 h, with MTT values consistently above 85%, indicating no cytotoxicity within the biologically relevant range. Concentrations above 4 µM were excluded because preliminary tests indicated reduced solubility and potential interference with the MTT reaction, and the doses shown in the figure were sufficient to elicit a clear biological response. (B) Dexamethasone (0.5–16 µM) reduced nitrite levels in a dose-dependent manner, reaching an IC₅₀ of 6.65 µM. A broader concentration range was applied because higher doses are required for this drug to exhibit measurable anti-inflammatory effects. (C) KW18 inhibited nitrite accumulation in a dose-dependent fashion, with a markedly lower IC₅₀ of 0.21 µM. As the curve demonstrated potent inhibition at the lowest concentrations plotted, extending the graph beyond 4 µM was unnecessary, since all relevant activity was captured within this effective range, and higher concentrations did not provide additional information.

Nitrite levels measured in the culture supernatants confirmed that LPS stimulation significantly increased nitric oxide production relative to untreated cells. As expected, dexamethasone (0.5–16 µM) reduced nitrite accumulation in a dose-dependent manner, with an IC₅₀ of 6.65 µM (Fig. 10B). In contrast, KW18 produced a markedly more substantial inhibitory effect, with an IC₅₀ of 0.21 µM (Fig. 10C). Notably, even the lowest concentrations tested (0.125–0.25 µM) suppressed nitrite production more effectively than the IC₅₀ of dexamethasone. These findings highlight KW18 as a potent anti-inflammatory molecule, supporting its potential therapeutic value, particularly considering its parallel antimicrobial efficacy against *S. aureus*, *P. aeruginosa*, *C. glabrata*, and *C. tropicalis*.

#### Cytokine profiling via ELISA

**Fig. 11:**
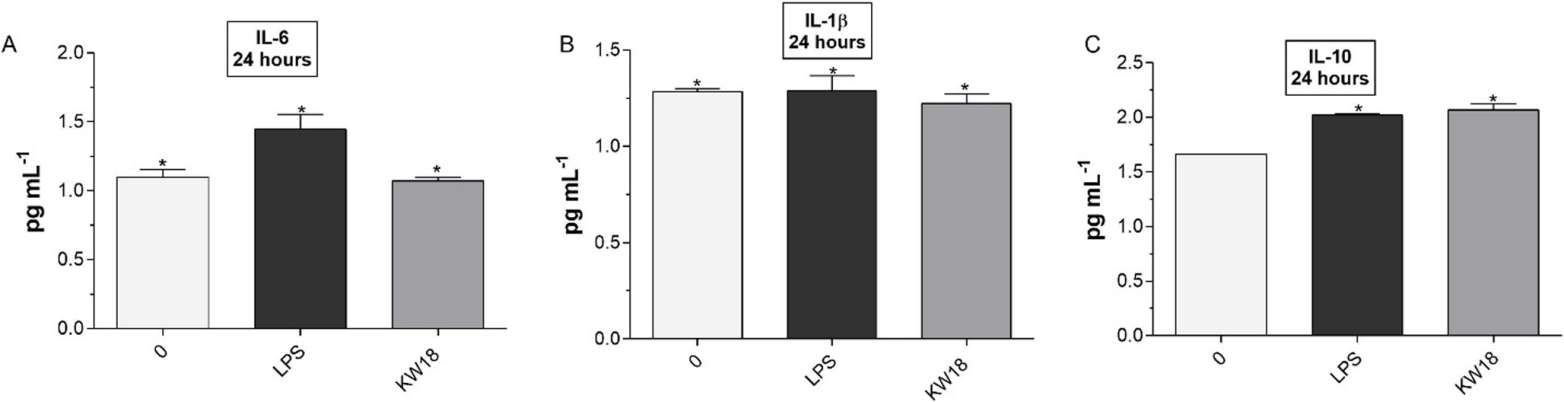
Impact of KW18 on cytokine secretion in BV-2 microglial cells following LPS stimulation. Cytokine concentrations in the supernatant were determined by ELISA. (**A**) KW18 treatment markedly reduced IL-6 levels compared to LPS alone. (**B**) No significant difference was observed in IL-1β levels among groups. (**C**) KW18 significantly increased IL-10 secretion relative to LPS. *p* < 0.05 indicates statistical significance versus the LPS group

To assess KW18’s immunomodulatory effects, we measured the secretion of pro-inflammatory cytokines (IL-6 and IL-1β) and the anti-inflammatory cytokine (IL-10) in BV-2 microglial cells after a 24-hour treatment, using enzyme-linked immunosorbent assay (ELISA). As shown in Fig. 11A, lipopolysaccharide (LPS) significantly increased IL-6 levels compared to the untreated control. By contrast, KW18 markedly reduced IL-6 secretion, indicating anti-inflammatory activity. Regarding IL-1β (Fig. 11B), no significant differences were observed among the control, LPS-stimulated, and KW18-treated groups, suggesting that KW18 does not notably affect IL-1β production in this setting. Importantly, KW18 modestly but significantly increased IL-10 levels compared to the LPS group (Fig. 11C), suggesting an immunoregulatory role that promotes inflammation resolution. These results indicate that KW18 influences microglial responses by decreasing IL-6 and increasing IL-10 levels.

Figure 11. Impact of KW18 on cytokine secretion in BV-2 microglial cells following LPS stimulation. ELISA was used to determine cytokine concentrations in the supernatant. (A) KW18 treatment markedly reduced IL-6 levels compared to LPS alone. (B) No significant difference in IL-1β levels was observed among groups. (C) KW18 significantly increased IL-10 secretion relative to LPS. *p* < 0.05 indicates statistical significance versus the LPS group.

## Discussion

AMPs are short amino acid sequences that combat multidrug-resistant pathogens through multiple mechanisms [7, 8]. Advances in peptide design have enhanced the stability and activity of peptides under physiological conditions, addressing limitations of natural AMPs, such as human β-defensin 3, which exhibits poor performance in high-salt or biofilm environments [10, 36]. The synthetic peptide KW18, rationally designed through bioinformatics optimization, integrates cationic, hydrophobic, and aromatic residues to confer broad-spectrum antimicrobial, antibiofilm, and immunomodulatory properties [15, 37]. Comprising 18 amino acids and approximately 1.98 kDa, KW18 is well-suited for short cationic peptides, facilitating membrane penetration and rapid action [10]. Positively charged lysine and arginine residues mediate electrostatic attraction to negatively charged microbial membranes [37], while hydrophobic and aromatic residues (tyrosine, tryptophan, and phenylalanine) facilitate membrane insertion and destabilization [36, 37].

NMR structural analysis demonstrated that KW18 adopts a stable amphipathic α-helix extending from residues Arg⁴ to Phe¹³, characterized by a distinct segregation of hydrophobic and cationic regions, an arrangement typical of membrane-active peptides [11, 13]. This α-helical conformation remains consistent across both solution and membrane environments, thereby favoring alignment along lipid bilayers and supporting a “carpet-like” mechanism of membrane disruption rather than pore formation [15]. The high structural convergence of the KW18 ensemble (backbone RMSD = 0.55 ± 0.17 Å) supports a well-defined a-helical architecture that optimally orients hydrophobic and cationic residues for membrane association [12, 17]. These features are consistent with stabilization patterns commonly observed in other amphipathic peptides [9, 18]. These structural determinants translate directly into the biological performance of KW18 [10, 15]. Its amphipathic α-helix and balanced charge–hydrophobicity ratio enable selective membrane targeting, leading to rapid bactericidal and fungicidal activity with minimal host toxicity [6, 9]. Pursuing more stable AMPs has led to the development of optimized constructs like KW18 [10, 38]. KW18 demonstrated potent activity against *S. aureus*, *P. aeruginosa*, *C. glabrata*, and *C. tropicalis*, even in media supplemented with ergosterol and sorbitol, indicating that its antimicrobial and antifungal mechanisms are likely independent of direct ergosterol binding [39]. The maintenance of antifungal efficacy in the presence of ergosterol, particularly against *C. tropicalis*, together with its tolerance to osmotic and ionic stress, suggests that KW18 acts primarily through non-specific membrane disruption rather than sterol-specific interactions [40]. The enhanced activity of KW18 against Gram-positive bacteria is mainly attributed to the absence of an outer membrane and the high exposure of negatively charged components, such as teichoic and lipoteichoic acids, in their cell wall [10, 39]. These features favor stronger electrostatic interactions with the cationic, amphipathic α-helical structure of KW18, leading to more efficient membrane disruption [7, 8, 36].

Another marked difference in susceptibility was observed between *Klebsiella pneumoniae* ATCC 700,603 and *Klebsiella oxytoca* ATCC 13,182, suggesting that intrinsic strain-specific characteristics significantly influence the interaction of these bacteria with the KW18 peptide [37]. This variation is likely associated with structural and physiological differences in the bacterial envelope [36]. In particular, *K. oxytoca* may possess a more rigid or densely cross-linked outer membrane, with variations in lipopolysaccharide (LPS) composition and surface charge that hinder the electrostatic interaction and membrane insertion of cationic peptides such as KW18 [10, 41]. Differences in the expression of major outer membrane porins (e.g., OmpK35 and OmpK36) may also contribute, since reduced porin expression limits permeability and decreases the peptide’s access to the inner membrane [35, 38]. Additionally, enhanced activity of efflux systems such as AcrAB-TolC could further reduce the intracellular accumulation of KW18, conferring a more resistant phenotype [33, 39]. Other factors, including differences in lipid composition, membrane fluidity, and the ability to form protective biofilm layers, may also play secondary roles in modulating peptide susceptibility [11, 41]. Taken together, these findings indicate that the observed variability between *Klebsiella* strains is not random but rather reflects the multifactorial and strain-dependent nature of bacterial resistance to cationic amphipathic peptides [13, 25, 37].

Compared with previously described synthetic antimicrobial peptides, such as WLBU2—which has been primarily characterized for its activity against *P. aeruginosa* and *A. baumannii* biofilms—KW18 expands the current understanding of peptide-based strategies by demonstrating activity across bacterial and fungal pathogens relevant to polymicrobial infections [40, 42]. Likewise, peptides such as SAAP-148 have been reported to exhibit potent antibiofilm activity against resistant bacterial species. However, their activity has been described primarily in a bactericidal context, with limited evidence of antifungal effects [40, 43]. In this broader framework, KW18 contributes additional insight by integrating both antibacterial and antifungal actions, supporting the growing interest in multifunctional peptides for complex infectious scenarios [36, 44]. By contrast, KW18 can degrade mature biofilms and maintain efficacy under osmotic stress conditions. Issues of degradation and toxicity limit the use of natural AMPs such as LL-37 and melittin, whereas synthetic peptides, including Mel4 and D-IK8, often require adjunctive combination [44–46]. KW18 exhibited rapid bactericidal activity, eliminating *S. aureus* within 30 min and *P. aeruginosa* within 45 min. This kinetic profile contrasts with the markedly slower response commonly observed for fluoroquinolones; for example, ciprofloxacin-treated biofilms frequently retain viable subpopulations and do not reach complete eradication even after 240 min, as reported in the literature [40, 47]. Moreover, KW18 surpasses amphotericin B against *C. tropicalis* (45 vs. 60 min) and shows comparable activity against *C. glabrata* (240 vs. 180 min).

KW18 demonstrated greater efficacy in biofilm eradication than in biofilm inhibition, primarily due to its structural and physicochemical properties that favor interaction with complex microbial communities [46, 48]. As an amphipathic and cationic α-helical peptide, KW18 possesses strong membrane-disruptive capabilities that enable it to penetrate the dense extracellular polymeric substance (EPS) of mature biofilms, which is typically composed of polysaccharides, proteins, lipids, and extracellular DNA [29, 32]. This matrix acts as a physical and chemical barrier that limits the diffusion and activity of many conventional antimicrobials, particularly during the early stages of biofilm formation [47]. However, the positive charge and amphipathic nature of KW18 facilitate electrostatic attraction to the negatively charged components of the biofilm matrix, promoting deeper peptide diffusion and direct contact with embedded microbial cells [9, 15]. In mature biofilms, microbial cells exhibit reduced metabolic activity and altered membrane composition, which often contribute to antimicrobial resistance [14, 48]. KW18’s ability to disrupt cell membranes and destabilize the structural integrity of the EPS enables it to overcome these defenses, leading to the disintegration of the biofilm architecture and the killing of dormant or slow-growing cells [24, 32]. In contrast, during the biofilm formation phase, microbial adhesion and EPS production are dynamic processes that may limit peptide access and binding efficiency, resulting in lower inhibitory activity compared to eradication [42, 46]. Therefore, KW18’s physicochemical features make it particularly effective against established biofilms, where direct disruption of microbial membranes and degradation of the biofilm matrix are the dominant mechanisms of action [10, 48].

Ciprofloxacin was selected as the primary comparator because of its broad spectrum against *S. aureus* and *P. aeruginosa* [49]. Amphotericin B exerts its antifungal activity primarily through direct binding to ergosterol, but its clinical use is limited by nephrotoxicity and other adverse effects [50]. Ciproflaxin works by inhibiting DNA gyrase and topoisomerase IV, but it takes over 240 min to kill bacteria and has no antifungal activity [51]. By contrast, KW18 acts faster and in a broader range, including antifungal [52]. While vancomycin is effective against Gram-positive bacteria, it has limitations in biofilm penetration and poses a risk of nephrotoxicity [49, 53]. KW18 rapidly eradicates *S. aureus* and *P. aeruginosa* within 30–45 min, reducing biofilm mass by 74.5% in *P. aeruginosa* and 96.5% in *C. glabrata*, outperforming traditional antibiotics [52]. In contrast, KW18 exhibits potent antibiofilm activity—eliminating 96.5% of *C. glabrata* and 93.3% of *C. tropicalis*—while maintaining its efficacy even under ergosterol-rich and osmotic stress conditions. These results suggest that KW18’s mechanism of action does not depend on direct ergosterol binding but rather involves general membrane disruption, contributing to its broad antifungal potential and improved safety profile [49, 51]. Its synergy with amphotericin B (FICI = 0.28 for *C. glabrata*, 0.365 for *C. tropicalis*) indicates potential for combination therapy to reduce toxicity [50]. Therefore, the KW18 peptide provides a versatile alternative (disrupting membranes rather than targeting receptors) with a lower risk of resistance [49, 52]. KW18 acts via a receptor-independent membrane-disruptive mechanism, confirmed by fluorescence microscopy and Sytox™ Green uptake [50]. This reduces the likelihood of resistance compared with intracellular-targeting agents such as ciprofloxacin, amphotericin B, and aminofungin peptide [46, 53].

Although Amphotericin B is recognized as a highly potent broad-spectrum antifungal agent, it was included in this study as both a reference compound and for synergistic combination assays to evaluate whether KW18 could enhance antifungal efficacy or allow dose reduction [50, 51]. Despite its effectiveness, the clinical use of Amphotericin B is often limited by significant nephrotoxicity and cytotoxicity at therapeutic doses [54]. Therefore, testing its combination with KW18 was strategically relevant, as the cationic and amphipathic nature of KW18 facilitates interactions with negatively charged fungal membrane components, potentially promoting Amphotericin B insertion and amplifying membrane disruption [10, 51]. This cooperative effect could enable lower effective concentrations of Amphotericin B, minimizing host toxicity while maintaining or improving antifungal potency [51]. Moreover, exploring such combinations is particularly pertinent in the context of drug-resistant fungal pathogens, such as *Candida glabrata* and *Candida tropicalis*, which represent significant clinical challenges and frequent opportunistic infections in immunocompromised melanoma patients—the central biological context of this study [8, 10, 16, 51]. Thus, Amphotericin B served not only as a benchmark antifungal but also as a mechanistic probe to assess the therapeutic potential of KW18 in combination regimens aimed at enhancing efficacy and reducing toxicity [50].

In addition to its antimicrobial properties, KW18 exhibits notable anti-inflammatory effects, especially in BV2 microglial cells, a recognized model for neuroinflammation [31]. Cytokines like IL-6 and IL-1β are crucial for defending against pathogens, but their excess can lead to tissue damage [34, 35]. KW18 reduces IL-6 and IL-1β levels while increasing IL-10, demonstrating a protective immunomodulatory effect [33, 55]. These actions are likely attributable to its structural features, including amphipathicity, hydrogen bonding capacity, and aromatic residues [51]. Such characteristics may facilitate interactions with toll-like receptors and other components of immune-related membranes [10, 52]. Therefore, KW18’s molecular design promotes membrane disruption and enhances anti-inflammatory and antimelanoma activity, offering dual therapeutic benefits [53, 55, 56].

Compared to other AMPs, KW18 displays consistent immunoregulatory behavior [54, 57]. LL-37, for instance, can trigger pro-inflammatory responses under specific microenvironments, and lactoferricin B inconsistently induces IL-10 [34, 54, 58]. By contrast, KW18 consistently downregulates IL-6 and upregulates IL-10 secretion, thereby contributing to the reestablishment of immune balance during infection, in line with the cytokine modulation patterns [35, 59]. In BV2 microglia, KW18 effectively inhibits NO production, as demonstrated by its low IC₅₀ value of 0.21 µM [33, 60]. This is significantly more effective than dexamethasone, a common anti-inflammatory drug, which has an IC₅₀ of 6.65 µM [61]. Moreover, KW18 treatment maintains high cell viability, with over 85% of cells remaining alive, indicating low toxicity [57, 61]. Compared with synthetic AMPs such as Mel4 (lacking immunomodulatory activity) and LTX-109 (high cytotoxicity), KW18 achieves both microbial clearance and balanced immune modulation [31, 35, 46]. KW18 is a promising therapeutic candidate due to its rapid, broad-spectrum antimicrobial activity, strong antibiofilm potential, low toxicity, and potent immunomodulatory effects [10, 52, 61]. By suppressing IL-6 and IL-1β, enhancing IL-10, and reducing NO production, KW18 exhibits dual antimicrobial and anti-inflammatory benefits [36, 55].

## Conclusion

The synthesized peptide KW18, developed through in silico rational design strategies, demonstrates potent broad-spectrum antimicrobial, antifungal, antibiofilm, and immunomodulatory properties. Its amphipathic structure enables rapid and non-specific disruption of microbial membranes across multiple pathogens, including resistant strains of *S. aureus*, *P. aeruginosa*, *C. tropicalis*, and *C. glabrata*. Unlike ergosterol-binding antifungals, KW18 retains its activity in ergosterol-supplemented and high-osmolarity environments, suggesting a broad-spectrum mechanism driven by membrane destabilization rather than specific sterol interaction. KW18 kills more rapidly than standard antibiotics and antifungals and works synergistically with them, indicating its potential for combination treatments that could lower dosage and reduce toxicity. Its capacity to both prevent and eliminate biofilms offers a significant advantage for treating chronic and device-associated infections. Mechanistic studies confirm membrane disruption as the primary antimicrobial mechanism, while immunomodulatory tests show it selectively reduces inflammation, evidenced by decreased IL-6 and increased IL-10 levels. Overall, these multifunctional features, low toxicity, and rapid response make the KW18 peptide an all-in-one candidate for developing therapies against multidrug-resistant infections and related inflammatory diseases. Future research should aim to optimize formulations and conduct in vivo tests to determine their clinical usefulness.

## Data Availability

No datasets were generated or analysed during the current study.
